# *ramR* mutations affecting fluoroquinolone susceptibility in epidemic multidrug-resistant *Salmonella enterica* serovar Kentucky ST198

**DOI:** 10.3389/fmicb.2013.00213

**Published:** 2013-07-31

**Authors:** Sylvie Baucheron, Simon Le Hello, Benoît Doublet, Etienne Giraud, François-Xavier Weill, Axel Cloeckaert

**Affiliations:** ^1^INRA, UMR1282 Infectiologie et Santé PubliqueNouzilly, France; ^2^Université François Rabelais de Tours, UMR1282 Infectiologie et Santé PubliqueTours, France; ^3^Institut Pasteur, Unité des Bactéries Pathogénes Entériques, Centre National de Référence des Escherichia coli, Shigella et SalmonellaParis, France

**Keywords:** *Salmonella*, ciprofloxacin resistance, efflux pump, regulation, ram

## Abstract

A screening for non-target mutations affecting fluoroquinolone susceptibility was conducted in epidemic multidrug-resistant *Salmonella enterica* serovar Kentucky ST198. Among a panel of representative isolates (*n* = 27), covering the epidemic, only three showed distinct mutations in *ramR* resulting in enhanced expression of genes encoding the AcrAB-TolC efflux system and low increase in ciprofloxacin MIC. No mutations were detected in other regulatory regions of this efflux system. Ciprofloxacin resistance in serovar Kentucky ST198 is thus currently mainly due to multiple target gene mutations.

## Introduction

Fluoroquinolones, together with extended-spectrum cephalosporins, are the treatment of choice for nontyphoid salmonellosis, as stable resistance to the most common members of different families of antimicrobial agents (ampicillin, chloramphenicol, streptomycin, sulfonamides, and tetracycline) has developed during the 1990s with the epidemic *Salmonella enterica* serovar Typhimurium phage type DT104 (Cloeckaert and Schwarz, 2001). Emerging resistance to fluoroquinolones in *Salmonella* spp. has been reported for both human and animal cases and is thus threatening to become a serious public health problem (Cloeckaert and Chaslus-Dancla, 2001; Piddock, 2002; Velge et al., 2005; Giraud et al., 2006). Of particular concern is the international spread of ciprofloxacin-resistant serovar Kentucky ST198 (Le Hello et al., 2011). This clone is not only highly resistant to ciprofloxacin but also multidrug-resistant (MDR) due to the presence of the *Salmonella* genomic island 1 (SGI1) carrying a multiple antibiotic resistance gene cluster, mostly variant SGI1-K carrying another resistance gene cluster (Doublet et al., 2008; Le Hello et al., 2011). SGI1 was initially identified in MDR serovar Typhimurium DT104 (Boyd et al., 2001), but nor the MDR serovar Typhimurium DT104 clone neither other MDR *S. enterica* serovars carrying SGI1 or variants of it, have to our knowledge been reported to display this high-level ciprofloxacin resistance.

In *Salmonella* spp., quinolone/fluoroquinolone resistance is mostly attributed to point mutations in the quinolone resistance-determining regions (QRDRs) of the target genes *gyrA, gyrB, parC*, and *parE*. For the *gyrA* gene, coding for the A subunit of DNA gyrase, mutations resulting in amino acid changes at Ser83 (to Phe, Tyr, or Ala) or at Asp87 (to Gly, Asn, or Tyr) are the most frequently observed in nalidixic acid-resistant strains (Cloeckaert and Chaslus-Dancla, 2001; Piddock, 2002; Velge et al., 2005; Giraud et al., 2006). High-level fluoroquinolone resistance has been reported in several *S. enterica* serovars (Choleraesuis, Schwarzengrund, Typhimurium) and is essentially due to the combination of several target gene mutations of which the most frequent are double mutations resulting in modifications of both residues 83 and 87 of GyrA together with one mutation leading to the amino acid change Ser80Ile in the ParC subunit of topoisomerase IV (Baucheron et al., 2002, 2004; Chu et al., 2005). In addition two main other mechanisms have been reported consisting of active afflux mediated by the chromosomally-encoded AcrAB-TolC efflux system and target protection by Qnr proteins which are mostly encoded by plasmids acquired by horizontal transfer (Giraud et al., 2006). However, according to the literature over 15 years, these mechanisms appear less frequently and thus from an epidemic point of view seem of lesser importance than multiple target gene mutations to reach high-level ciprofloxacin resistance and compromise treatment.

In the case of ciprofloxacin resistance in serovar Kentucky ST198, three combinations of multiple target modifications, acquired in a possible sequential way, have been reported consisting of a first GyrA Ser83Phe modification, followed by three different situations of a second GyrA modification at position 87, i.e., Asp87Asn, Asp87Gly, or Asp87Tyr, and finally the ParC modification Ser80Ile (Le Hello et al., 2011). Qnr proteins have not been reported yet as additional mechanism for this epidemic clone, and active efflux has been suspected in a previous study due to a moderate increase of production in some isolates of the AcrA protein belonging to the AcrAB-TolC efflux system (Weill et al., 2006).

In the present study we assessed the frequency of enhanced efflux by AcrAB-TolC in a subset of serovar Kentucky ST198 strains of the 2000–2005 period of the epidemic. In case of significant increased production of AcrAB-TolC we investigated more deeply the regulatory mechanisms behind this overproduction, in particular the involvement of the *ram, sox*, and *mar* regulatory loci (Abouzeed et al., 2008; Kehrenberg et al., 2009). Among these loci, the *ramRA* locus appears to be the most important in regulating AcrAB-TolC expression in *Salmonella* spp. (Abouzeed et al., 2008; Kehrenberg et al., 2009). *ramR* encodes a repressor protein (RamR) belonging to the TetR family of repressor proteins, and has been shown to be the local repressor protein of *ramA* transcription (Abouzeed et al., 2008; Baucheron et al., 2012); while *ramA* encodes a transcriptional activator protein (RamA) belonging to the AraC/XylS family of regulatory proteins (Nikaido et al., 2008). The latter is involved in upregulating expression of the AcrAB-TolC system (Nikaido et al., 2008). Several mutations in *ramR* or its binding site upstream of *ramA*, affecting expression of this efflux system, have been detected in clinical isolates of serovar Typhimurium and of minor serovars Hadar, Infantis, Livingstone, or Schwarzengrund (Abouzeed et al., 2008; Kehrenberg et al., 2009; Hentschke et al., 2010; Akiyama and Khan, 2012).

## Materials and methods

The 27 serovar Kentucky ST198 strains selected for this study are shown in Table 1. Bacterial isolates were selected for this study, based on their evolutionary history following the emergence of target gene mutations initially in *gyrA* at the commencement of the epidemic in 2000–2002, followed by isolates with additional mutations (in *gyrA* and *parC*) toward the end in 2002–2005 and which demonstrated a higher MIC toward ciprofloxacin. An additional criterion for selection consisted of the differences observed in ciprofloxacin MICs suggestive for another resistance mechanism than target gene mutation. MICs were determined as described previously (Baucheron et al., 2002, 2004). SGI1 detection and characterization were performed as described previously (Boyd et al., 2001; Doublet et al., 2008). Efflux pump production was assessed by Dot blot using an anti-AcrA polyclonal antibody as described previously (Abouzeed et al., 2008). Occurrence of mutations affecting *acrAB* and *tolC* expression was determined by PCR and sequencing the regulatory regions *ramR-ramA, acrR-acrA, marC*-marO-*marR-marA, soxS-soxR*, and *acrS-acrE* using primers listed in Table 2. Transcription levels of *ramA, acrA*, and *tolC* were determined by qRT-PCR as described previously (Giraud et al., 2013).

**Table 1:** ***Salmonella enterica* serovar Kentucky ST198 strains analyzed in this study**.

**Strain**	**Country**	**Year of isolation**	**Antimicrobial resistance profile**	**SGI1**	**PFGE type**	**CIP MIC (μg/ml)**	**Substitution(s) in the QRDR of:**		**Mutation(s) in efflux pump regulatory regions**	**AcrA production ratio**
							**GyrA**	**ParC**		
00 1059	Egypt	2000	AMX NAL	+ (SGI1-P1)	XKEN-1a	0.125	S83F	None		3
01 2100	Egypt	2001	AMX STR SPT GEN SUL TET NAL	+ (SGI1-K1)	XKEN-1a	0.125	S83F	None	–	2
02 2818	Egypt	2002	AMX STR SPT GEN SUL TET NAL	+	XKEN-1i	0.5	S83F	None	+ *(ramR)*	5
02 2691	Egypt	2002	AMX STR SPT GEN SUL TET NAL	+ (SGI1-K3)	XKEN-1a	0.125	S83F	None	–	1
02 8051	Egypt	2002	AMX STR SPT GEN SUL TET NAL	+	XKEN-1a	0.25	S83F	None	–	1
02 8141	Egypt	2002	AMX STR SPT GEN SUL TET NAL	+ (SGI1-K1)	XKEN-1m	0.5	S83F	None	+ (*ramR*)	5
02 9866	Egypt	2002	AMX STR SPT GEN SUL TET NAL CIP	+	XKEN-1a	8	S83F, D87N	S80I	–	2
03 9270	India	2003	NAL	–	XKEN-2d	0.125	S83F	None	–	1
04 2049	Egypt	2004	NAL CIP	+	XKEN-1b	8	S83F, D87G	S80I	–	2
04 4567	Egypt	2004	AMX STR SPT GEN SUL TET NAL CIP	+ (SGI1-K1)	XKEN-1g	4	S83F, D87G	S80I	–	2
04 6248	Egypt	2004	STR SPT GEN SUL TET NAL CIP	+	XKEN-1a	8	S83F, D87G	S80I	–	1
04 7734	Egypt	2004	AMX STR SPT GEN SUL TET NAL	+ (SGI1-K1)	XKEN-1h	0.5	S83F	None	–	1
04 8262	Egypt	2004	STR SPT GEN SUL NAL CIP	+ (SGI1-K5)	XKEN-1a	8	S83F, D87N	S80I	–	1
04 9384	Egypt	2004	AMX STR SPT GEN SUL TET NAL CIP	+	XKEN-1g	4	S83F, D87G	S80I	–	1
05 0490	Egypt	2005	STR SPT GEN SUL TET NAL CIP	+	XKEN-1a	4	S83F, D87G	S80I	–	2
05 0520	Egypt	2005	AMX NAL CIP	+ (SGI1-P2)	XKEN-1a	4	S83F, D87Y	S80I	–	1
05 1016	Kenya	2005	NAL CIP	+ (SGI1-Q2)	XKEN-1a	4	S83F, D87Y	S80I	–	1
05 1199	Egypt	2005	STR SPT GEN SUL NAL CIP	+ (SGI1-Q3)	XKEN-1a	4	S83F, D87G	S80I	–	1
05 2131	Egypt	2005	AMX NAL CIP	+ (SGI1-Q1)	XKEN-1a	4	S83F, D87N	S80I	–	3
05 2354	Kenya/ Tanzania	2005	AMX STR SPT GEN SUL TET NAL CIP	+	XKEN-1c	8	S83F, D87Y	S80I	–	3
05 3290	Egypt	2005	AMX STR SPT GEN SUL TET NAL CIP	+	XKEN-1c	4	S83F, D87G	S80I	–	1
05 3883	Kenya/ Tanzania	2005	AMX STR SPT GEN SUL TET NAL CIP	+	XKEN-1d	4	S83F, D87Y	S80I	–	2
05 4680	Sudan	2005	STR SPT GEN SUL TET NAL CIP	+ (SGI1-K4)	XKEN-1l	4	S83F, D87G	S80I	–	2
05 7714	Unknown	2005	AMX NAL CIP	+	XKEN-1b	4	S83F, D87N	S80I	–	2
05 8560	Tunisia	2005	AMX STR SPT GEN SUL TET NAL CIP	+	XKEN-1d	16	S83F, D87N	S80I	+ (*ramR*)	6
05 236	Egypt	2005	AMX NAL CIP	+	XKEN-1c	4	S83F, D87N	S80I	–	1
05 5111	Libya	2005	AMX SUL TET NAL CIP	+ (SGI1-K2)	XKEN-1a	4	S83F, D87N	S80I	–	1

**Table 2:** **Primers used for PCRs**.

**Primer used and target region**	**Primer**	Nucleotide position relative to the LT2 strain genome^*^	**Oligonucleotide sequences(s) (5′ to 3′)**	**Size (bp)**	**Annealing temp (°)C**	**References**
**DETECTION OF MUTATIONS**						
*ramR-ramA*	ram5	638085	TCGGTAAAAGGCAGTTCCAG	958	60	This study
	ramA6	639042	GTCGATAACCTGAGCGGAAA			
*acrR-acrA*	acrR1	533463	CAGTGGTTCCGTTTTTAGTG	992	58	Olliver et al., 2005
	acrR2	534454	ACAGAATAGCGACACAGAAA			
*marC*-marO*-marR-marA*	marR1	1597459	CAGTGTTGCGTCTGGACATC	787	60	This study
	marR2	1598245	GCTAACGGGAGCAGTACGAC			
*soxS-soxR*	sox1	4503970	CTACAGGCGGTGACGGTAAT	915	60	This study
	sox2	4504884	CGGCGCTTTAGTTTTAGGTG			
*acrS-acrE*	acrS1	3560054	TTGGCATTAATTGCCTCACA	1094	62	This study
	acrS2	3561128	ATGATGAATGAGGGCAGGAG			
**qRT-PCR**						
*gmk*	gmk-f	3933294	TTGGCAGGGAGGCGTTT	62	60	Baucheron et al., 2012
	gmk-r	3933355	GCGCGAAGTGCCGTAGTAAT			
*gyrB*	gyrB-f	4040275	TCTCCTCACAGACCAAAGATAAGCT	81	60	Baucheron et al., 2012
	gyrB-r	4040195	CGCTCAGCAGTTCGTTCATC			
*rrs*	rrs-f	NA^**^	CCAGCAGCCGCGGTAAT	57	60	Baucheron et al., 2012
	rrs-r	NA^**^	TTTACGCCCAGTAATTCCGATT			
*ramA*	ramA-f	639180	GCGTGAACGGAAGCTAAAAC	167	60	Baucheron et al., 2012
	ramA-r	639346	GGCCATGCTTTTCTTTACGA			
*acrA*	acrA-f	533120	GAAACCGCACGTATCAACCT	220	60	Baucheron et al., 2012
	acrA-r	532901	CCTGTTTCAGCGAACCATTT			
*tolC*	tolC-f	3349107	GCCCGTGCGCAATATGAT	67	60	Baucheron et al., 2012
	tolC-r	3349173	CCGCGTTATCCAGGTTGTTG			

* GenBank NC_003197.1.

** NA: Not Applicable due to the number of copies of this gene in Salmonella.

## Results and discussion

**Figure 1:**
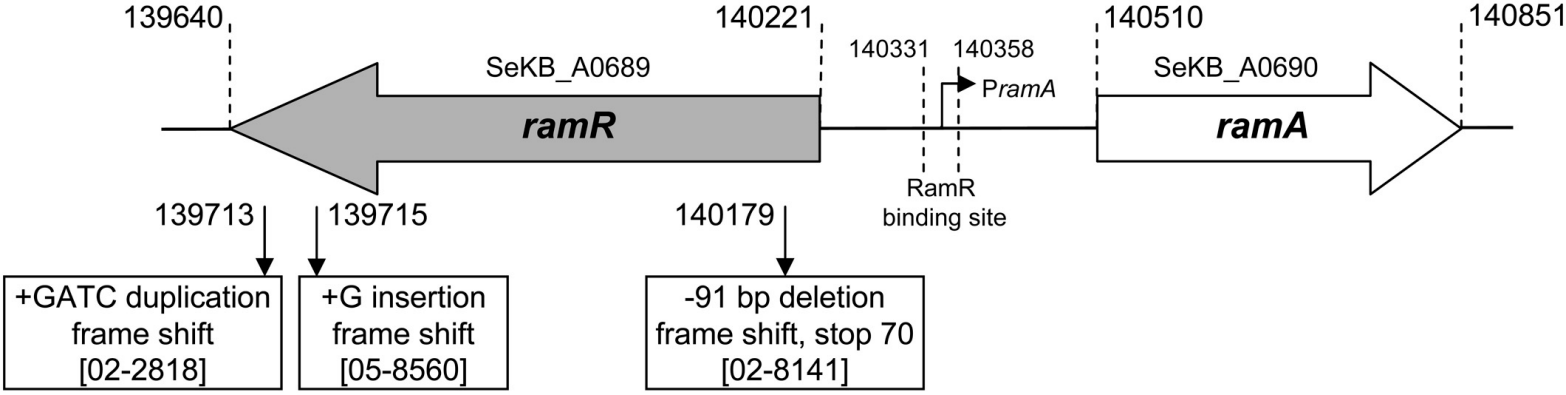
**Mutations detected in *the ramR*-*ramA* region relative to the genome sequence of *S. enterica* serovar Kentucky strain CDC 191 (GenBank: ABEI01000007.1)**.

As shown in the Table 1 most of the strains selected carried SGI1 or variants of it and were thus MDR. They were all from human cases in France who acquired their infection during travel to Africa or India. As assessed by Dot blot, most of the strains (*n* = 24) did not show significant increased production of AcrA relative to susceptible serovar Kentucky reference strain 98K (AcrA production ratios from 1 to 2; Table 1). Relative to strain 98K, three strains showed a 3-fold increased AcrA production, and more suggestive for increased active efflux three strains a 5- to 6-fold increased production of AcrA (Table 1). Among these regulatory regions, mutations were detected only in the *ramR* open reading frame and in only three strains of this study (Table 3). The mutations were distinct frame shift mutations and consisted of a GATC duplication for strain 02-2818, a G insertion for strain 05-8560, and a 91 bp deletion for strain 02-8141 (Figure 1). The role of these mutations in upregulating *acrAB* and *tolC* expression, and consecutive enhanced efflux-mediated resistance, was further assessed by: (i) complementing with the wild-type *ramR* gene (using plasmid pRamR Abouzeed et al., 2008); (ii) determining the MICs of ciprofloxacin and unrelated antibiotic florfenicol shown to be substrate of AcrAB-TolC (Baucheron et al., 2002); and (iii) measuring expression of *ramA, acrA*, and *tolC* by qRT-PCR (Giraud et al., 2013). The results shown in Table 3 are in agreement with data published previously for other *S. enterica* serovars (Abouzeed et al., 2008; Kehrenberg et al., 2009), i.e. *ramR* mutations observed account for a 2- to 4-fold increased resistance level by active efflux through enhanced expression of AcrAB-TolC. As also observed in previous studies, the effect of such mutations on *ramA* transcription level was significantly higher than on *acrA or tolC* transcription levels. It is somehow expected considering the direct local repressor activity of RamR on *ramA* transcription and the distant RamA transcriptional activator activity on *acrAB* and *tolC* (Abouzeed et al., 2008; Baucheron et al., 2012; Giraud et al., 2013).

**Table 3:** **Characteristics of the *Salmonella enterica* serovar Kentucky ST198 strains carrying *ramR* mutations**.

**Strain**	**Source**	**Geographic origin**	**Antimicrobial resistance profile**^**a**^	**PFGE type**	**SGI1 (variant)**^**b**^	**MIC of indicated antibiotic (μg/ml)**			**Substitution(s) in the QRDR of:**		**Mutation in *ramR***	**Transcription levels of:**		
						**NAL**	**CIP**	**FFC**^**c**^	**GyrA**	**ParC**		***ramA***	***acrA***	***tolC***
**MDR STRAINS**														
05-8560	Human	Tunisia	AMX STR SPT GEN SUL TET NAL CIP	XKEN-1d	+ (Ks)	>1024	16	16	S83F, D87Y	S80I	1 bp insertion (position 506)	24.6	7.2	2.6
05-8560(pRamR)						>1024	4	4				2.4	1.7	1.7
02-8141	Human	Egypt	AMX STR SPT GEN SUL TET NAL	XKEN-1m	+ (K1)	512	0.50	16	S83F	–	91 bp insertion (position 42)	106.1	10.4	7.8
02-8141(pRamR)						512	0.125	8				1.6	1.1	1.2
02-2818	Human	Egypt	AMX STR SPT GEN SUL TET NAL	XKEN-1i	+ (Ks)	512	0.50	16	S83F	–	4 bp duplication (position 508)	29.1	5.3	4.7
02-2818(pRamR)						256	0.25	4				1.9	0.9	1.6
02-9866	Human	Egypt	AMX STR SPT GEN SUL TET NAL CIP	XKEN-1a	+ (Ks)	>1024	8	4	S83F, D87N	S80I	–	2.9	1.2	1.6
02-9866(pRamR)						>1024	4	4				1.8	1.6	2.4
**REFERENCE STRAIN**														
98K	Chicken	USA	Susceptible	XKEN-4	–	1	0.004	2	–	–	–	1.0	1.0	1.0
98K(pRamR)						1	0.004	2				2.1	1.3	1.5

a AMX, amoxycillin; STR, streptomycin; SPT, spectinomycin; GEN, gentamicin; SUL, sulfonamides; TET, tetracycline; NAL, nalidixic acid; CIP, ciprofloxacin.

b Ks: subgroup of SGI1-K.

c FFC, florfenicol.

Non-target mutations as assessed in this study confirm they are infrequent in *Salmonella* spp. but seem nevertheless mostly restricted to the *ram* regulatory region. Most mutations in the *ramR*-*ramA* region reported to date, as also shown in this study, are distinct and found in single isolates. To our knowledge only independent isolates of the epidemic ciprofloxacin-resistant serovar Typhimurium DT204 clone from the 1990s have been shown to carry the same mutation in *ramR* consisting of an insertion by an IS*1* element (Abouzeed et al., 2008). We may nevertheless expect that the further global spread of ciprofloxacin-resistant serovar Kentucky ST198 and its resistance evolution will possibly, like in the case of serovar Typhimurium DT204, result in successful *ramR*-mutation-carrying subclones.

### Conflict of interest statement

The authors declare that the research was conducted in the absence of any commercial or financial relationships that could be construed as a potential conflict of interest.
